# Supplementary material for: Single-cell somatic copy number variants in brain using different amplification methods and reference genomes
Source: bioRxiv. 2023 Aug 8:2023.08.07.552289. Preprint. [Version 1] doi: 10.1101/2023.08.07.552289 (PMC10441336; doi:10.1101/2023.08.07.552289)
Supplement: Supplementary file 1 [file NIHPP2023.08.07.552289v1-supplement-3.pdf]

bioRxiv preprint doi: https://doi.org/10.1101/2023.08.07.552289; this version posted August 8, 2023. The copyright holder for this preprint (which was not certified by peer review) is the author/funder, who has granted bioRxiv a license to display the preprint in perpetuity. It is made available under aCC-BY 4.0 International license.

Supplementary information

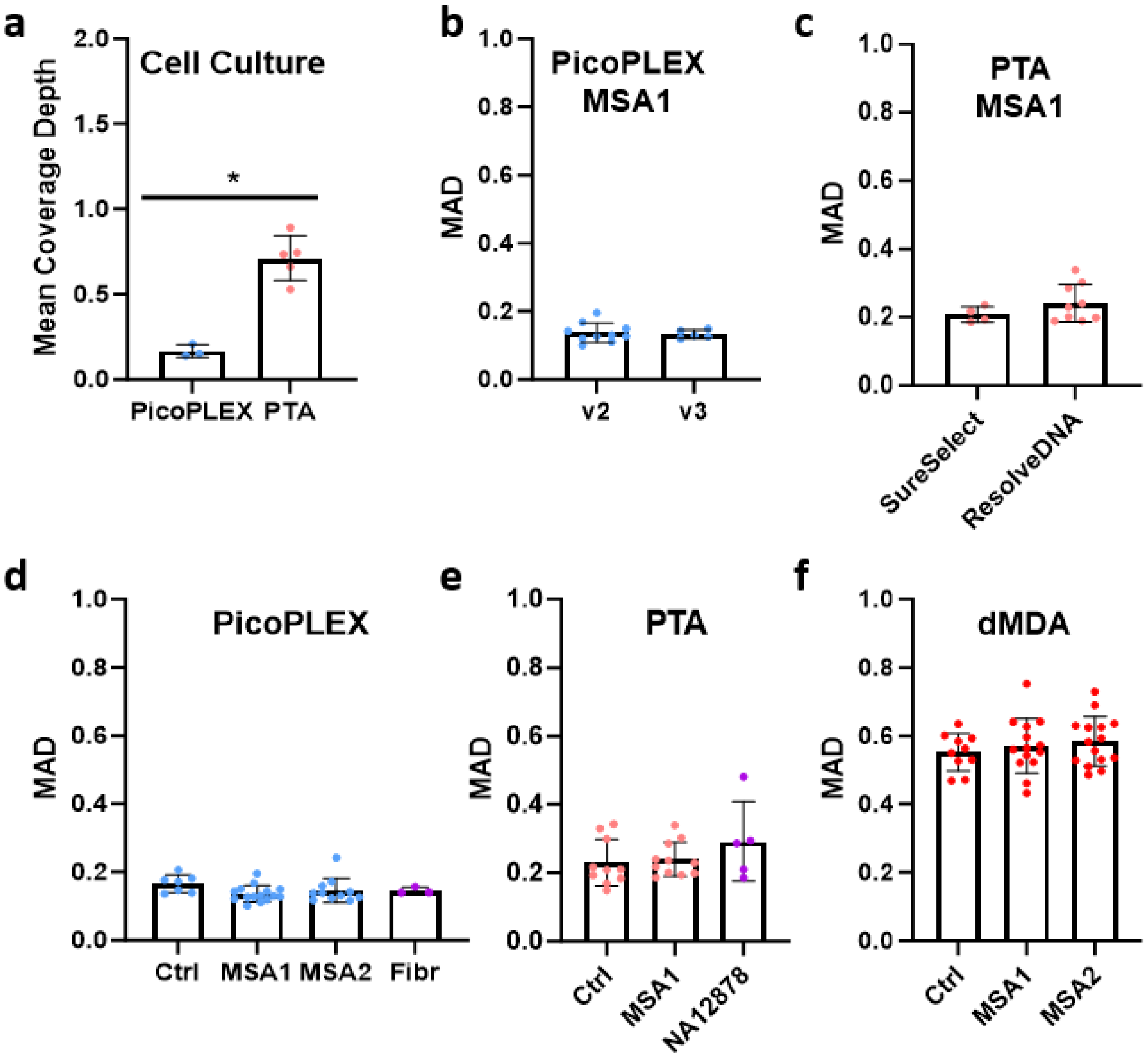

Supplementary Fig. 1 Sequencing coverage of non-brain samples and detailed MAD comparison between samples and method variations.

a Mean coverage depth distribution of nuclei from cell culture amplified by either PicoPLEX (fibroblasts with known *SNCA* triplication isolated by CellRaft, *n*=3) and PTA (NA12878 cells, isolated by nuclei sorting, *n*=5) *; *p*=0.04 unpaired Mann-Witney test. **b-f** Median Absolute Deviation (MAD scores) of nuclei aligned to hg38 at 500 kb bins.

b MAD scores PicoPLEX amplified nuclei from MSA1 donor amplified by either PicoPLEX v2 (Takara R300672, *n*=10) or PicoPLEX v3 (Takara R300722, *n*=5) analyzed by unpaired Mann-Whitney test. v2 vs v3 ns (*n*=0.95).

c MAD from PTA-amplified brain nuclei when used either SureSelect (Agilent) or ResolveDNA (BioSkryB) libraries analyzed by unpaired Mann-Whitney test. SureSelect vs ResolveDNA ns (*n*=0.33).

bioRxiv preprint doi: https://doi.org/10.1101/2023.08.07.552289; this version posted August 8, 2023. The copyright holder for this preprint (which was not certified by peer review) is the author/funder, who has granted bioRxiv a license to display the preprint in perpetuity. It is made available under aCC-BY 4.0 International license.

d PicoPLEX-amplified nuclei from brain donors (Ctrl *n*=7, MSA1 *n*=15, MSA2 *n*=11) or fibroblast (*n*=3) cells analyzed by Kruskal-Wallis test with Dunn's multiple comparisons test Ctrl vs MSA1 ns (adj. *p*=0.09), Ctrl vs MSA2 ns (adj. *p*=0.28), Ctrl vs Fibr ns (adj. *p*>0.99), MSA1 vs MSA2 ns (adj. *p*>0.99), MSA1 vs Fibr ns (adj. *p*>0.99), MSA2 vs Fibr ns (adj. *p*>0.99).
e PTA-amplified nuclei from different brain donors (Ctrl *n*=10), MSA1 *n*=11) or NA12878 cells (*n*=5) analysed by Kruskal-Wallis test with Dunn's multiple comparisons test. Ctrl vs MSA1 ns (adj. *p*>0.99), Ctrl vs NA12878 ns (adj. *p*=0.88), MSA1 vs NA12878 ns (adj *p*>0.99).
f MAD of dMDA-amplified from different brain donors (Ctrl *n*=10), MSA1 *n*=14, MSA2 *n*=15) analysed by Brown-Forsythe and Welch ANOVA tests with Dunnett's T3 multiple comparisons tests. All comparisons ns (adj. p>0.99).
Data represent Mean ± SD.

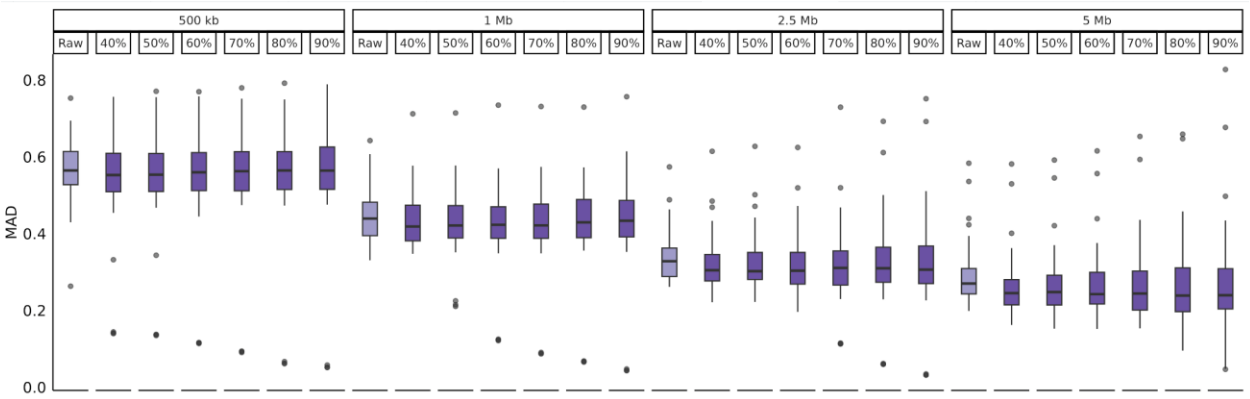

Supplementary Fig. 2 MAD of dMDA at different bin sizes.
The results are shown before denoising ("raw") and after denoising at different proportions. For each bin size, we tested the difference between the raw data and after denoising (40%) using the Wilcoxon test with "paired=TRUE" and alternative="greater". For all bin sizes, except for 500 kb (*p*=0.06), we found that 40% denoising significantly improved the MAD score: for bin sizes 1 Mb, 2.5 Mb, and 5 Mb, the *p*-value was < 0.01.

bioRxiv preprint doi: https://doi.org/10.1101/2023.08.07.552289; this version posted August 8, 2023. The copyright holder for this preprint (which was not certified by peer review) is the author/funder, who has granted bioRxiv a license to display the preprint in perpetuity. It is made available under aCC-BY 4.0 International license.

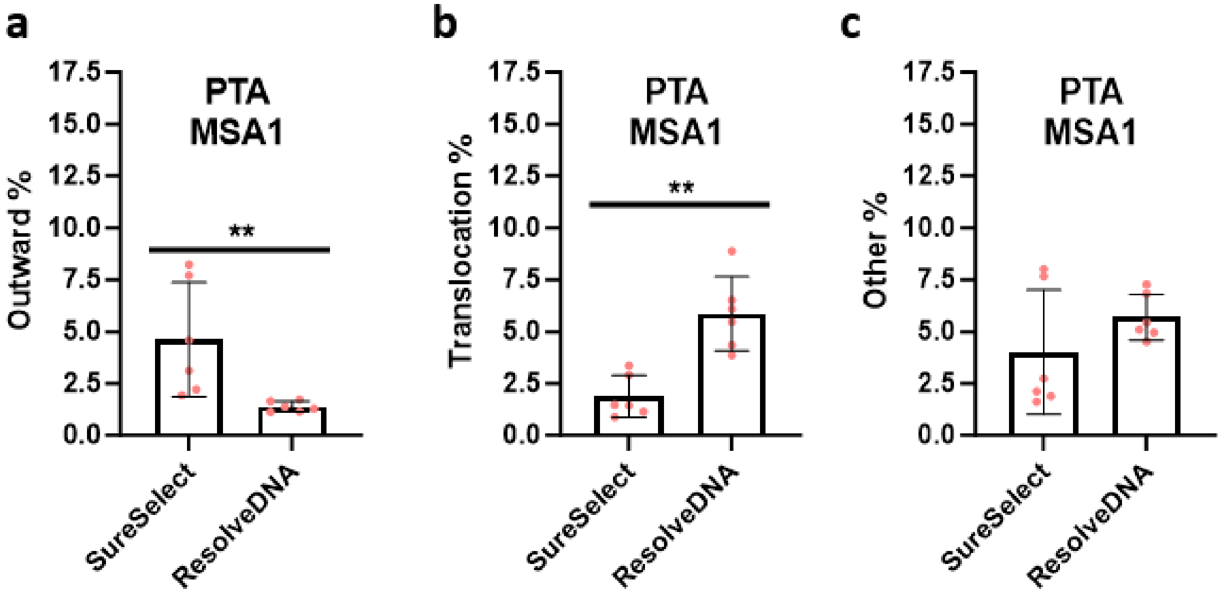

Supplementary Fig. 3 Effect of different library preparations for PTA samples on discordant read pair orientation.

PTA-amplified cells from MSA1 brain sequenced before either SureSelect (n=6) or ResolveDNA (n=6) library preparation. Analyzed by Mann–Whitney, unpaired student's t-test, mean ± SD shown.

a Outward pairs. SureSelect vs ResolveDNA ** (p=0.002).

b Translocations. SureSelect vs ResolveDNA ** (p=0.002).

c Other. SureSelect vs ResolveDNA ns (p=0.39).

bioRxiv preprint doi: https://doi.org/10.1101/2023.08.07.552289; this version posted August 8, 2023. The copyright holder for this preprint (which was not certified by peer review) is the author/funder, who has granted bioRxiv a license to display the preprint in perpetuity. It is made available under aCC-BY 4.0 International license.

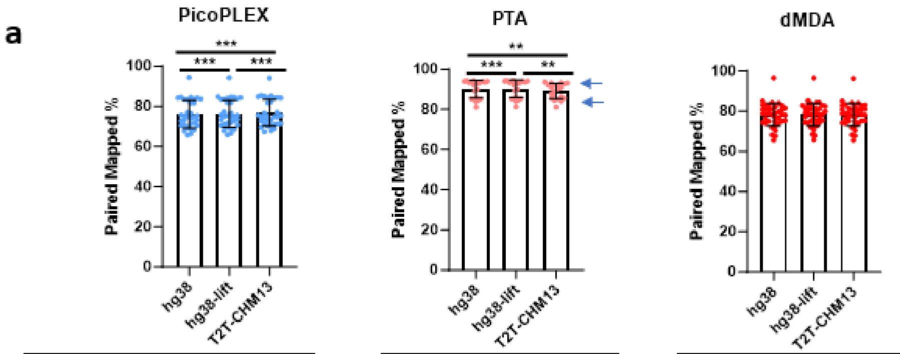

	hg38	hg38-lift	T2T-CHM13
Mean	76.16	76.35	76.77
SD	6.86	6.81	6.64

hg38	hg38-lift	T2T
90.17	90.27	89.15
4.40	4.35	3.76

hg38	hg38-lift	T2T
78.16	78.28	78.19
5.65	5.67	5.61

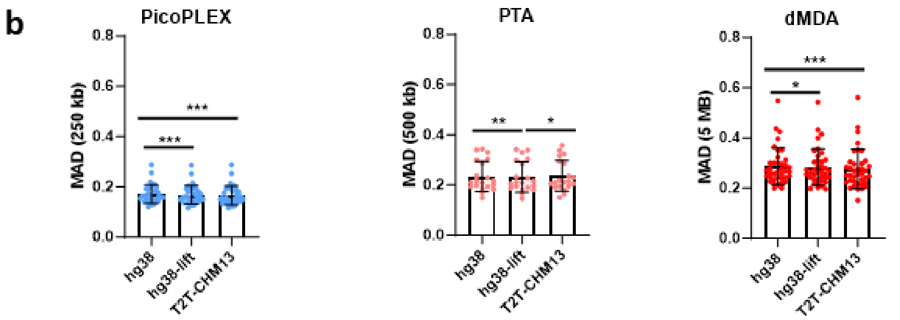

	hg38	hg38-lift	T2T-CHM13
Mean	0.171	0.168	0.166
SD	0.036	0.036	0.037

hg38	hg38-lift	T2T-CHM13
0.235	0.232	0.236
0.060	0.061	0.061

hg38	hg38-lift	T2T-CHM13
0.286	0.283	0.276
0.072	0.072	0.078

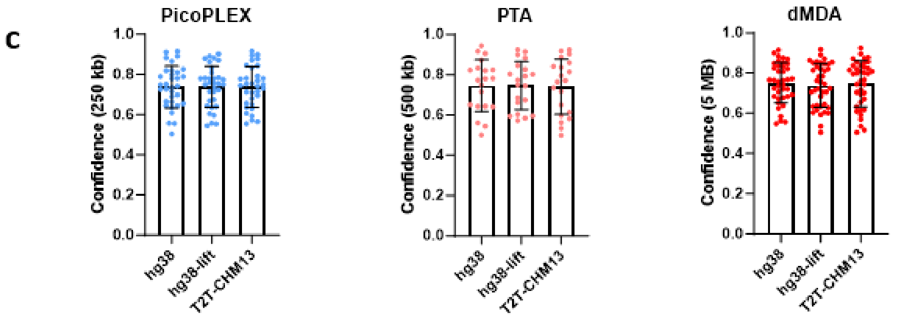

	hg38	hg38-lift	T2T-CHM13
Mean	0.738	0.739	0.738
SD	0.104	0.102	0.102

hg38	hg38-lift	T2T-CHM13
0.747	0.747	0.741
0.129	0.118	0.138

hg38	hg38-lift	T2T-CHM13
0.751	0.738	0.747
0.100	0.109	0.114

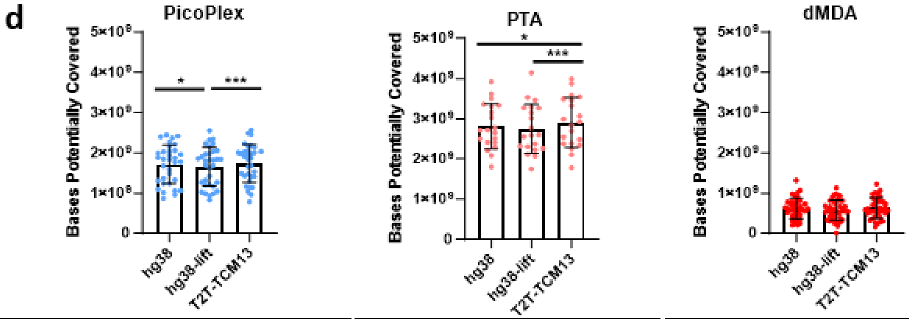

	hg38	hg38-lift	T2T-CHM13	hg38	hg38-lift	T2T-CHM13	hg38	hg38-lift	T2T-CHM13
Mean	1,707,393,939	1,644,826,699	1,735,333,333	2,814,000,000	2,751,575,182	2,900,500,000	615,447,368	573,131,311	624,763,158
SD	476,714,861	482,163,937	465,718,240	564,935,022	610,780,493	622,951,168	252,818,886	250,881,179	253,839,642

(Continues on the next page)

bioRxiv preprint doi: https://doi.org/10.1101/2023.08.07.552289; this version posted August 8, 2023. The copyright holder for this preprint (which was not certified by peer review) is the author/funder, who has granted bioRxiv a license to display the preprint in perpetuity. It is made available under aCC-BY 4.0 International license.

Supplementary Fig. 4 Single-cell metrics across different reference genomes.

a % of reads aligned. RM one-way ANOVA with the Geisser-Greenhouse correction and Tukey's multiple comparisons test. PicoPLEX (*n*=33): all comparisons *** (*p*<0.001). PTA (*n*=21): arrows indicate library prep; top=ResolveDNA (n=12), bottom=SureSelect (n=9). hg38 vs hg38-lift ***(*p*<0.001), hg38 vs T2T-CHM23 **(*p*=0.007), hg38-lift vs T2T-CHM23 ** (*p*=0.003). dMDA (*n*=39) : hg38 vs hg38-lift ns (*p*=0.14), hg38 vs T2T-CHM23 ns (*p*=0.27), hg38-lift vs T2T-CHM23 ns (*p*=0.32).

b MAD. Friedman test with Dunn's multiple comparisons test. PicoPLEX (n= 33): hg38 vs hg38-lift ***(p<0.001), hg38 vs T2T-CHM23 ***(p<0.001), hg38-lift vs T2T-CHM23 ns(p=0.42). PTA (n=20): hg38 vs hg38-lift **(p=0.005), hg38 vs T2T-CHM23 ns(p>0.99), hg38-lift vs T2T-CHM23 *(p=0.03). dMDA (n= 38): hg38 vs hg38-lift *(p=0.02), hg38 vs T2T-CHM23 ***(p<0.001), hg38-lift vs T2T-CHM23 ns (p=0.26).

c Confidence scores. RM one-way ANOVA with the Geisser-Greenhouse correction and Tukey's multiple comparisons test. PicoPLEX (*n*=33): all comparisons ns, hg38 vs hg38-lift (*p*=0.98), hg38 vs T2T-CHM23 (*p*>0.99), hg38-lift vs T2T-CHM23 (*p*=0.95), PTA (*n*=20): all comparisons ns, hg38 vs hg38-lift (*p*>0.99), hg38 vs T2T-CHM23 (*p*=0.85), hg38-lift vs T2T-CHM23 (*p*=0.75), dMDA (n=38): all comparisons ns, hg38 vs hg38-lift (*p*=0.18), hg38 vs T2T-CHM23 (*p*=0.92), hg38-lift vs T2T-CHM23 (*p*=0.77).

d PreSeq. RM one-way ANOVA with the Geisser-Greenhouse correction and Tukey's multiple comparisons test. PicoPLEX (*n*=33): hg38 vs hg38-lift *(*p*=0.04), hg38 vs T2T-CHM23 ns(p=0.51), hg38-lift vs T2T-CHM23 ***(p<0.001), PTA (*n*=20): hg38 vs hg38-lift ns (*p*=0.06), hg38 vs T2T-CHM23 *(*p*=0.05), hg38-lift vs T2T-CHM23 ***(*p*<0.001), dMDA (*n*=38): all comparisons ns, hg38 vs hg38-lift (*p*=0.11), hg38 vs T2T-CHM23 (*p*=0.80), hg38-lift vs T2T-CHM23 (*p*=0.10).

Data represent Mean ± SD.

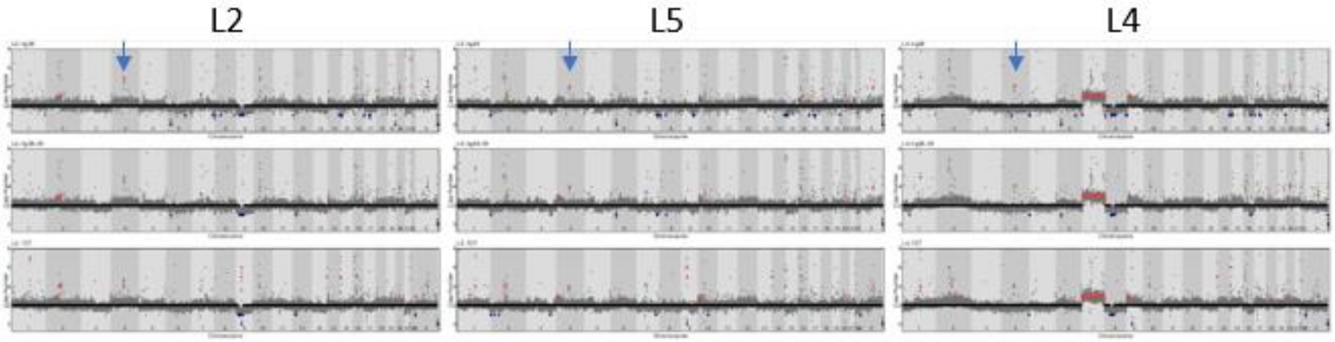

Supplementary Fig. 5 *SNCA* germline triplication (copy number 4) in three single fibroblasts.

PicoPLEX WGA, 250 kb bins. Arrow points to CNV. Reference genomes from top to bottom: hg38, liftover, T2T. Note that cell L2 has copy number assigned as 5 in the hg38 and liftover, although visual inspection indicates that the bin copy numbers are between 4 and 5. Cell L4 fails confidence score (0.6). Cell L4 also has a chromosome 7 gain.

bioRxiv preprint doi: https://doi.org/10.1101/2023.08.07.552289; this version posted August 8, 2023. The copyright holder for this preprint (which was not certified by peer review) is the author/funder, who has granted bioRxiv a license to display the preprint in perpetuity. It is made available under aCC-BY 4.0 International license.

Supplementary Fig. 6 CN profile of cells passing QC.
Each page shows the profile of one cell, including MAD, confidence score, bin size, and CNV calls. All CNVs called by *Ginkgo* are shown in the Table above the CN plot. Filtered CNVs are highlighted (pink = gain, blue = loss).

(file uploaded separately)

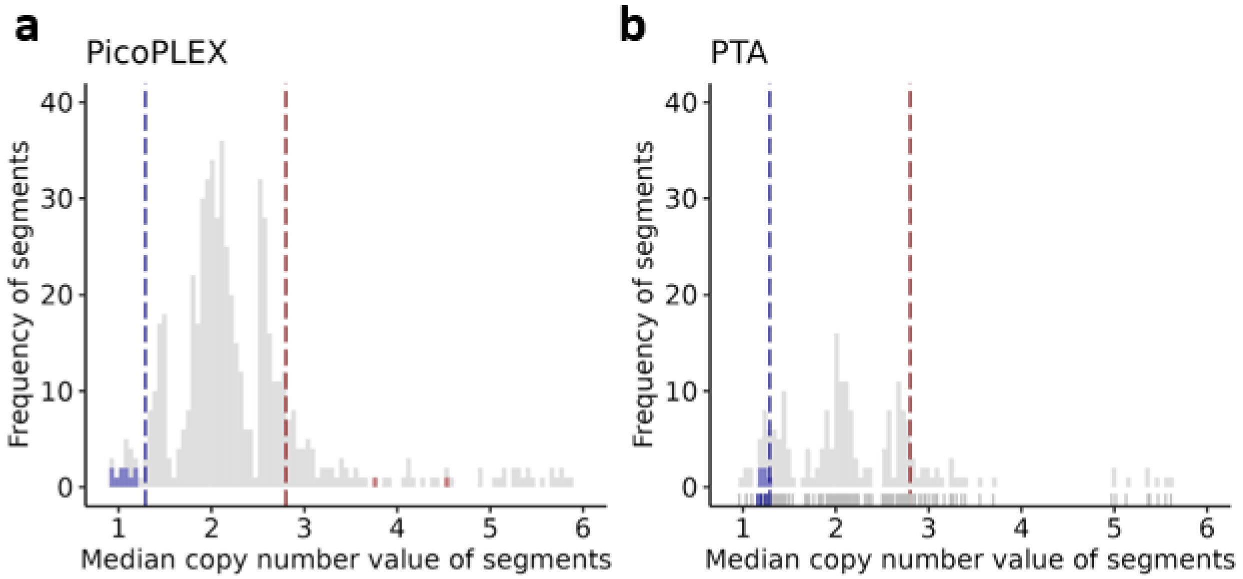

Supplementary Fig. 7 Determination of thresholds for CNV filtering.
Plots show the median CN of segments in cells passing QC. A. PicoPLEX. For losses, we set a threshold of 1.29 (dotted purple line), as the median CN of chromosome X was lower than that in all males (*n*=11). For gains, as the CN 2.8 was found in more segments than the immediately higher and lower values, we used that as the cut-off (dotted red line). The two segments in red are the *SNCA* CNV in the fibroblasts passing QC. B. PTA. As the data were sparse, we applied the same thresholds as in PicoPLEX, visually shown as purple and red lines. The median CN of chromosome X in males was below 1.29 in all 5 cells.

bioRxiv preprint doi: https://doi.org/10.1101/2023.08.07.552289; this version posted August 8, 2023. The copyright holder for this preprint (which was not certified by peer review) is the author/funder, who has granted bioRxiv a license to display the preprint in perpetuity. It is made available under aCC-BY 4.0 International license.

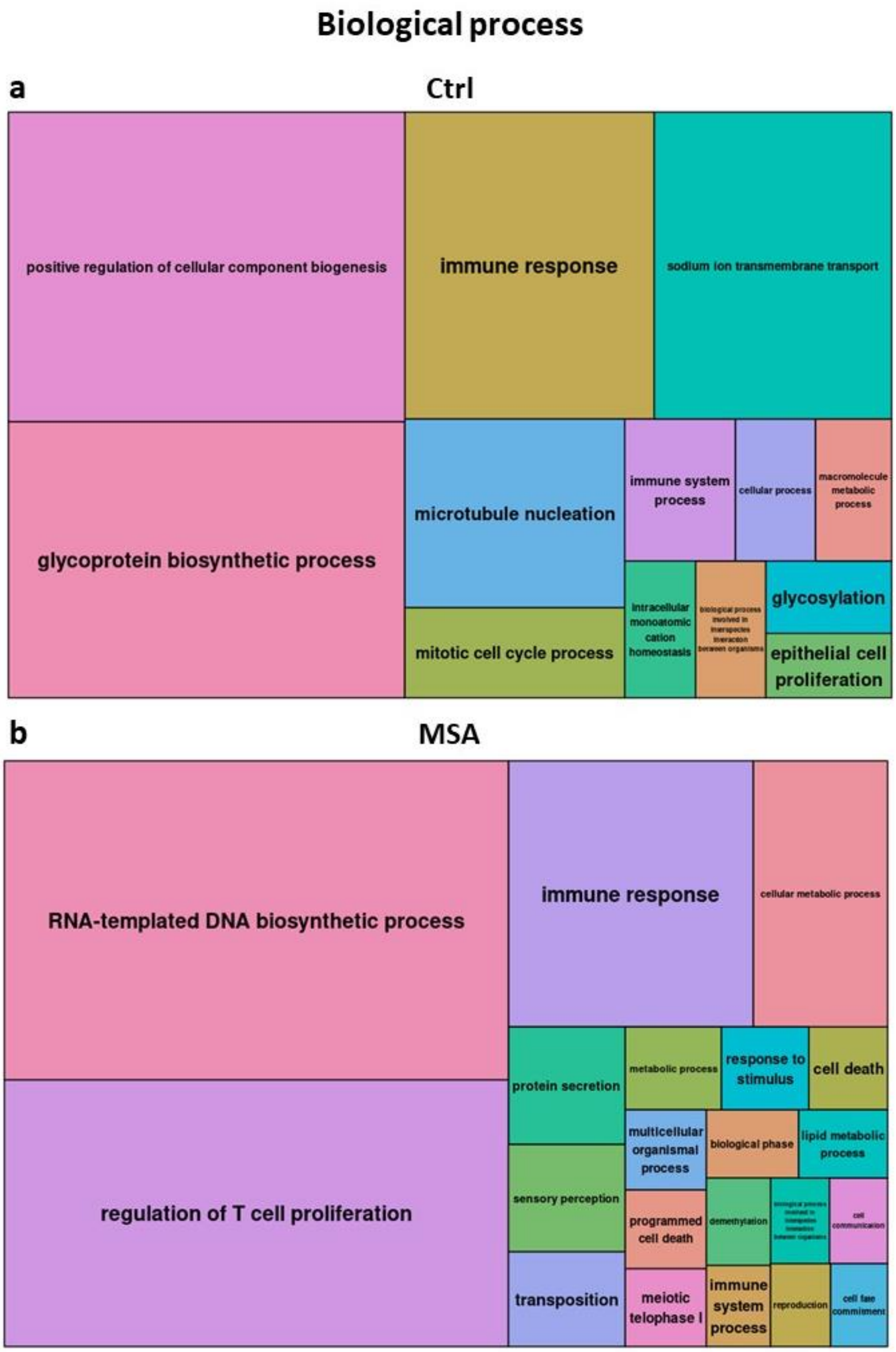

(Continues on the next pages)

bioRxiv preprint doi: https://doi.org/10.1101/2023.08.07.552289; this version posted August 8, 2023. The copyright holder for this preprint (which was not certified by peer review) is the author/funder, who has granted bioRxiv a license to display the preprint in perpetuity. It is made available under aCC-BY 4.0 International license.

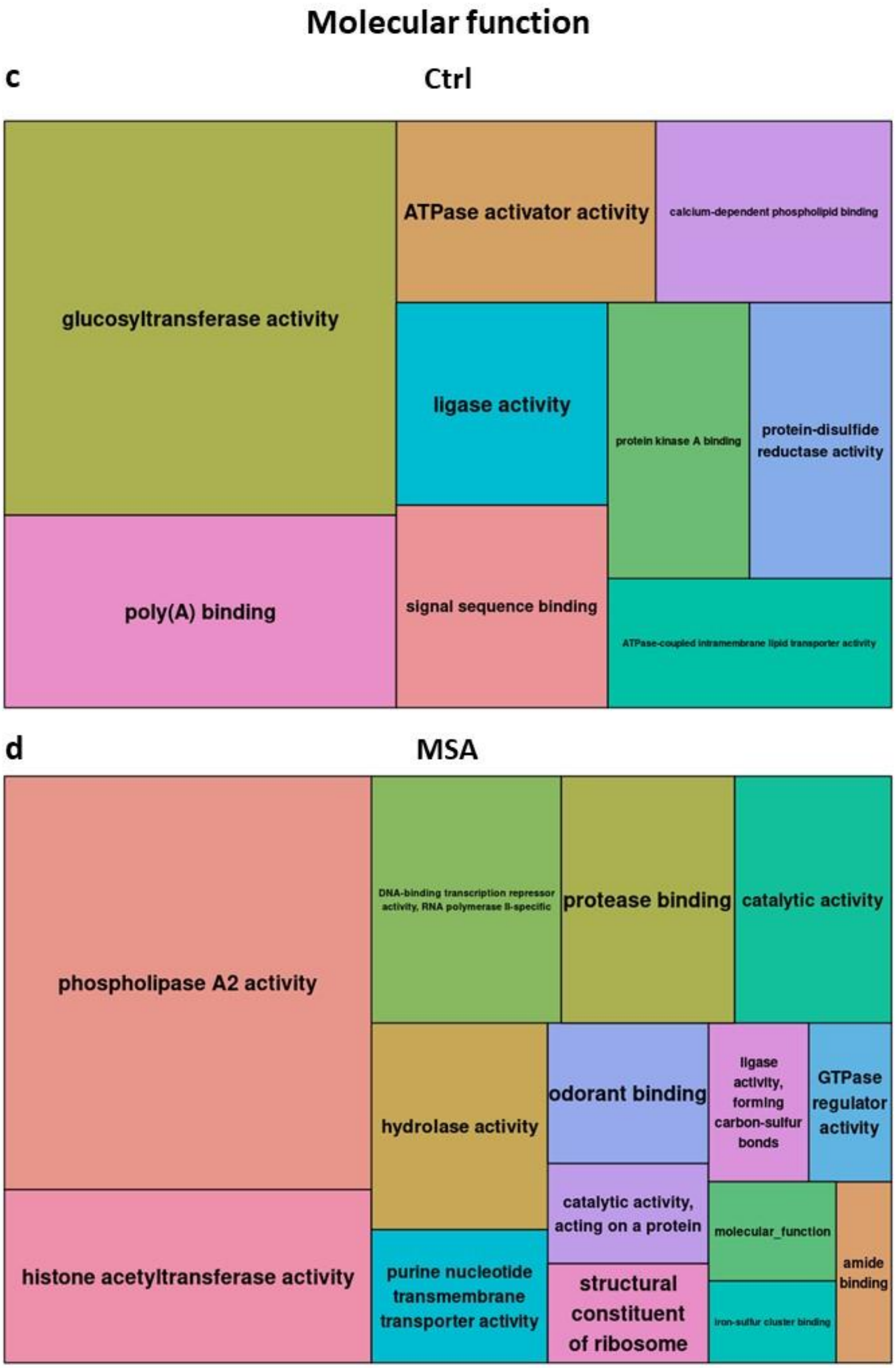

(Continues on the next page)

bioRxiv preprint doi: https://doi.org/10.1101/2023.08.07.552289; this version posted August 8, 2023. The copyright holder for this preprint (which was not certified by peer review) is the author/funder, who has granted bioRxiv a license to display the preprint in perpetuity. It is made available under aCC-BY 4.0 International license.

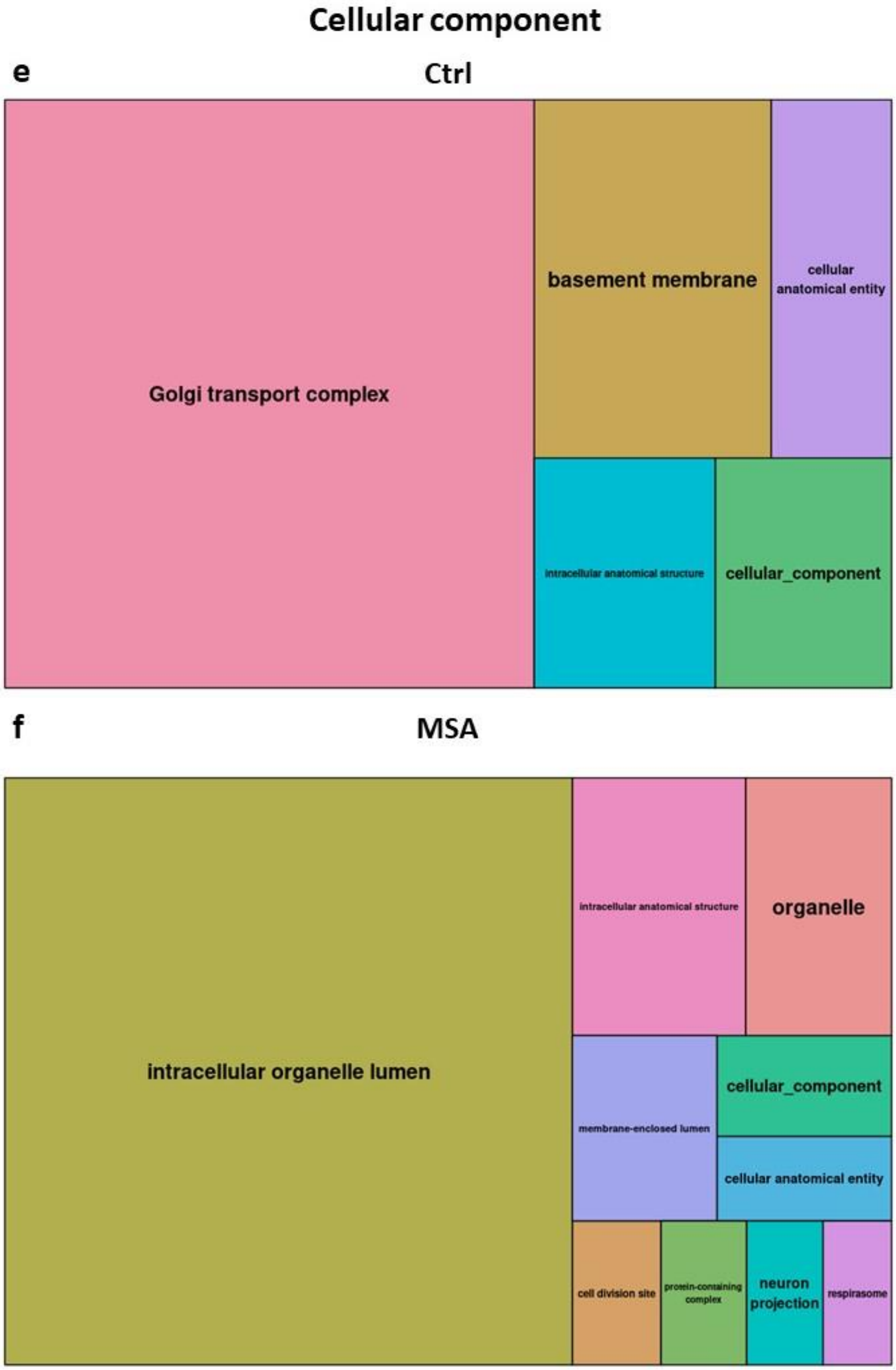

Supplementary Fig. 8 Gene ontology analysis.
a-b biological process for **a** control and **b** MSA brains, **c-d** molecular function for **c** control and **d** MSA brains. **e-f** cellular component for **e** control and **f** MSA brains.

bioRxiv preprint doi: https://doi.org/10.1101/2023.08.07.552289; this version posted August 8, 2023. The copyright holder for this preprint (which was not certified by peer review) is the author/funder, who has granted bioRxiv a license to display the preprint in perpetuity. It is made available under aCC-BY 4.0 International license.

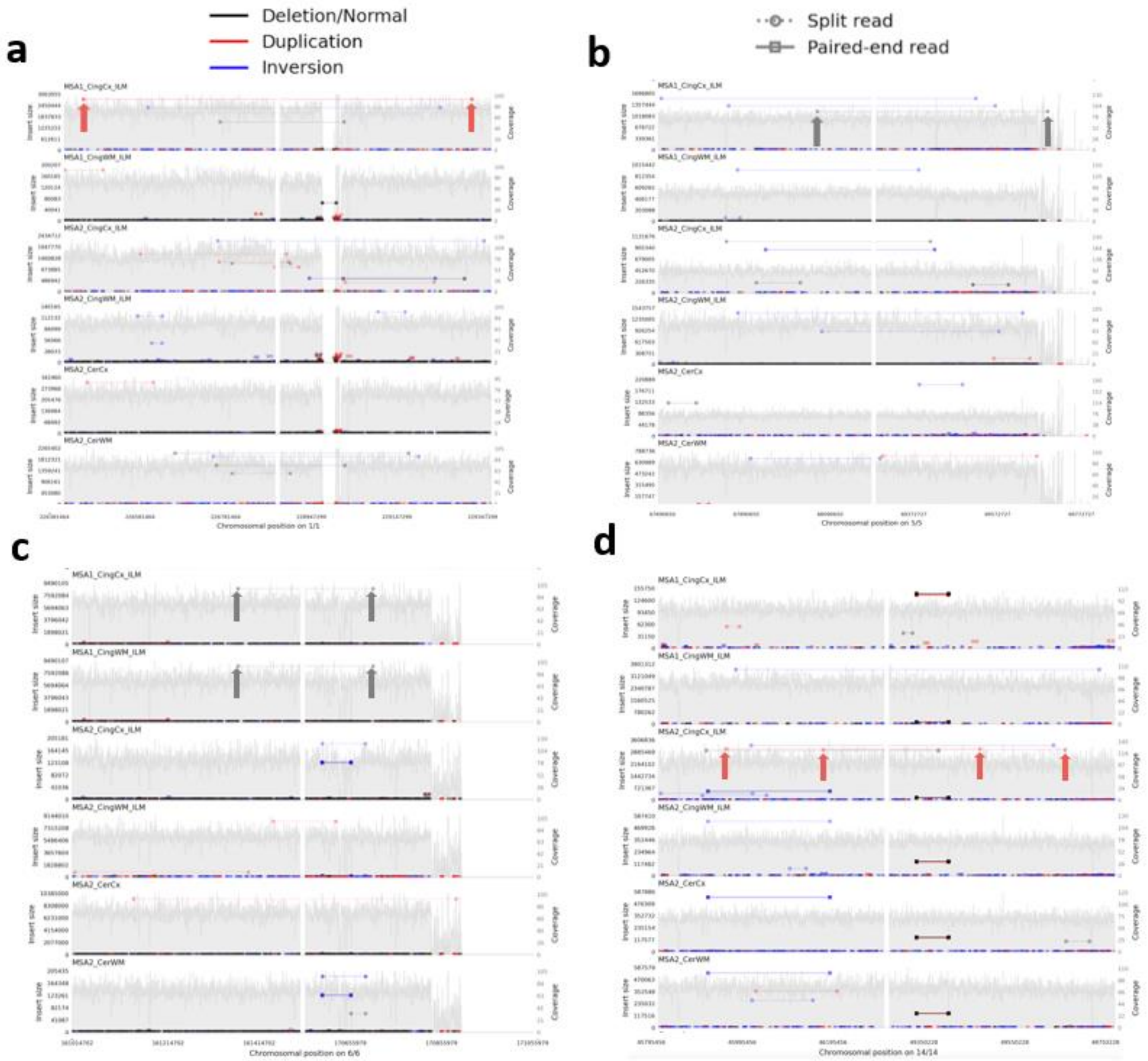

Supplementary Fig. 9 Samplot visualization of bulk Illumina (ILM) WGS in all brain samples focusing on genomic regions of single-cell CNVs supported by bulk reads.

All MSA1 and MSA2 brain regions with data available are shown; CingCx= cingulate cortex (also shown in **Fig. 4**), CingCx= cingulate white matter, CerCx= cerebellar cortex, CerWM= cerebellar WM. Read pairs supporting a particular type of CNV / SV indicated according to the scheme at the top, and those supporting each called single-cell CNV arrowed. The left and right panel of each plot show the regions around the reported proximal and distal breakpoint respectively, which is at the middle of each panel, with the chromosomal positions on the x axis below. The y axis indicates the calculated insert size for the read pairs of interest on the left, and the local coverage on the right. Note that each supporting read pair is only seen in the cingulate cortex of the MSA brain where the relevant single-cell was reported, except for **c** which is also shown in the adjacent white matter of the same brain.

bioRxiv preprint doi: https://doi.org/10.1101/2023.08.07.552289; this version posted August 8, 2023. The copyright holder for this preprint (which was not certified by peer review) is the author/funder, who has granted bioRxiv a license to display the preprint in perpetuity. It is made available under aCC-BY 4.0 International license.

Supplementary Tables

Supplementary Table 1 CNV calls (unfiltered) for PicoPLEX and PTA data across different genomes

	Gains / cell	Losses / cell
hg38	12.62	11.26
hg38-lift	14.41	4.00
T2T	16.98	3.40

Supplementary Table 2 Information About Significant CNVs:
This shows detailed information about all CNVs which were called after filtering. CN=copy number. Ginkgo results are shown in columns F-L, and Copykit (if called) L-O. The size difference for CNV called by both is shown in P.

(File uploaded separately)

Supplementary Table 3 Coverage of bulk WGS

Sample	Cingulate cortex	Cingulate White matter	Cerebellar cortex	Cerebellar white matter
MSA1	85.1	86.3		
MSA2	84.1	87.2	77.5	81.3
